# The bactericidal effect of an ionizer under low concentration of ozone

**DOI:** 10.1186/s12866-016-0785-5

**Published:** 2016-07-30

**Authors:** Jin-Soo Park, Bong-Jo Sung, Kyung-Soo Yoon, Choon-Soo Jeong

**Affiliations:** 1 Department of Biological Science, University of Ulsan, Ulsan, 44610 South Korea; 2 L&E Research Center, LG Electronics, Seoul, South Korea

**Keywords:** Ionizer, Low concentration of ozone, Negative and positive ions, Bactericidal effect, ROS

## Abstract

**Background:**

Several mechanisms have been suggested for the bactericidal action of ionizers including electrical phenomena, effects of negative and positive ions and electrostatic repulsion. Negative and positive ions have indeed been shown to have bactericidal effects. In addition, since ozone is generated along with ions, these may contribute to the bacterial killing. In this study, we used a newly developed ionizer, which generates a relatively low concentration of ozone, to determine whether its effect on bacterial cells were due to ions or ozone, and, if ions, how the ions exerted their effects.

**Results:**

The effect of ions on bacterial killing was compared with that of the ozone produced using an ion trap to remove the ions. The ionizer had the ability to kill the bacteria, and ion capture dramatically reduced its bactericidal effect, indicating that the ozone generated had little or no bactericidal effect under these conditions, and the ions produced were responsible for almost all the bacterial killing. Operation of the ionizer increased the level of 8-oxo-dG, a marker of oxidative DNA damage, and decreased aconitase activity, which is known to be sensitive to ROS. The ionizer further affected the adenylate energy charge of bacterial cells. Removal of the ions with the ion trap greatly reduced all these effects.

**Conclusion:**

These results indicate that negative and positive ions generated by the ionizer are responsible for inducing oxidative stress and so reducing bacterial survival.

**Electronic supplementary material:**

The online version of this article (doi:10.1186/s12866-016-0785-5) contains supplementary material, which is available to authorized users.

## Background

Negative and positive ions have been shown to have bactericidal effects on various bacterial species and fungi [1–5]. Recently, air ionizers have been installed to prevent bacterial infections during operations [6] and infections of items of plastic medical equipment [7]. Although, many studies indicate that negative and positive ions have bactericidal effects, the exact mechanisms involved remain unknown. Also some have argued that the bactericidal effects of ionizers are either overestimated [3] or due to ozone [8] or physical effects [7].

Oxidative stress is defined as a disturbance of the prooxidant/antioxidant balance in favor of prooxidants, leading to potential damage to the cell [9]. Excess prooxidants result in oxidative stress, which damages cell components such as lipids, proteins and DNA by oxidation [10, 11]. Oxidative stress-induced free radicals are highly reactive elements such as reactive oxygen species (ROS), which can attack biological molecules and lead to death.

A DC voltage applied to an ion generator will create a corona discharge at the end of the electrodes forming both ions and ozone. The amounts of ozone generated vary with the generator, and its effects on bacteria also differ [8]. In this study, we used an ion generator, which generates a relatively low concentration of ozone, with the aim of clarifying whether its effect on bacterial cells was due to ions or ozone, and, if ions, how ions exerted their effects.

## Methods

### Generating air ions

**Fig. 1:**
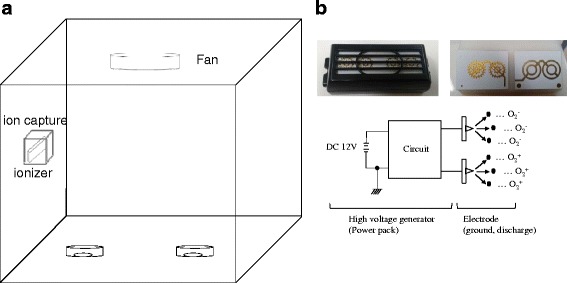
The experimental set-up and the ionizer. **a** The configuration and size of the test chamber and the locations of the ionizer and fan. The ionizer is located 30 cm from the bottom of the box, and the fan is located 7 cm from the top. **b** The ionizer has three components: high voltage generator (power pack), ground electrode and discharge electrode. It is characterized by a specific frequency and an alternate D.C. pulse-high positive/negative voltage. The input voltage of the power pack is 12 V in D.C., and the output voltage is peak-to-peak ± 2.8 kV

The ionizers (Plasmaster 2.5G) were supplied by LG Electric Company (Seoul, Korea). Ionizers are air ion generators governed by the principle of the corona discharge, which is used to create positive and negative air ions (Fig. 1). The corona discharge is an electrical discharge brought on by ionization of the air surrounding an electrode. It occurs when a sufficiently high voltage is supplied between the electrodes (ground and discharge). Then the discharge develops near the electrode (discharge) in the high field region and spreads out towards the other electrode (ground). Positive and negative air ions are produced simultaneously in the discharge region by electron-particle collisions and fusions.

### Bacterial strains and culture conditions

*Escherichia coli* ATCC 23736*, Staphylococcus aureus* ATCC 25923*, Bacillus subtilis* ATCC 6633, and *Enterococcus faecalis* ATCC 19433 were used. Cultures were grown on LB at 37 °C in a shaking incubator at 200 rpm for 16 h.

### Exposure to negative and positive ions

Ion exposure experiments were performed in a sealed plastic chamber (46*46*46 cm, 100 L) at room temperature and 60–70 % relative humidity (RH). Temperature and RH were monitored with Thermo Recoder TR-72Ui (T&D Co, Japan). Cultures were serially diluted with diluent (1:100 LB), and deposited by suction onto 0.45 μm membrane filters (Φ47mm, Porafil, Germany) with a vacuum pump (Gast, USA) for 10 s. They were placed in empty Petri-dishes without lids, and then exposed to the fan (12 V, 1.5 m/s, Cooler Master, Taipei) only (F mode), the fan plus ionizer (IZ + F mode), or the fan plus ionizer and ion capture (IZ + F + C mode) for 30 ~ 120 min. In the IZ + F + C mode, the input to the ionizer was adjusted to 14 V to achieve the same level of ozone production, while it was 12 V in IZ + F mode (discussed in Results). After exposure, the membranes were incubated on NA agar plates for 48 h at 37 °C.

The experiments were performed with two sets of filters; one containing ~10^3^ cfu/filter for percent survivals less than 10 %, usually exposed for 60–120 min, and the other containing ~10^2^ cfu/filter for percent survivals exceeding 10 %, usually exposed for 30–60 min. Control filters were placed on NA plate immediately after suction and incubated. Survival was measured by colony count. For experiments on sessile bacteria on agar plates, we used Φ60mm plates, on which the number of bacteria would not exceed 100 cfu/plate, to maintain RH in the chamber below 75 %. Concentrations of ions were measured with an Air Ion Counter (Model: COM-3400, COM Systems Inc., Japan). For ion measurements the ion monitor was installed at the bottom of the chamber at three points alongside the membrane filters.

### Ozone quantification

Ozone concentrations were determined by both an ozone meter (OZ 2000G), and the indigo method. The ozone meter was used to determine the concentration of ozone in the chamber, and the indigo method to make sure that the ozone reached the same concentration in conditions with and without ion capture. In the case of the ozone meter, the air sampler was placed at the bottom of the chamber and the air was sampled every 15 min for 2 h. For the indigo method, 12.5 μM of potassium indigotrisulfonate (Sigma, Inc., St Louis, MO, USA) solution was exposed in a 60Φ dish with stirring. Decolorization of the blue potassium indigotrisulfonate solution by dissolved ozone was measured with a SpectraMax M2 multi-microplate reader (Molecular Devices) at 600 nm.

### Ion capture

Ions were eliminated with an ion collector. Its principle is similar to that of electrostatic precipitation, which is a technique for removing particles suspended in air using an electrostatic force. The electric ion collector is composed of a low-voltage power supply and a collecting electrode. The input voltage of the power pack is 12 V in DC (Direct Current). Four wire-mesh layers are used for the collecting electrode; when positive and negative air ions pass through the wire mesh they are collected on the electrodes by applied negative and positive high voltages.

### ROS assay

Intracellular reactive oxygen species (ROS) were measured using an intracellular ROS assay kit (Cell Biolabs, Inc., San Diego, CA, USA). Cultured bacteria were incubated with 100 μM 2′, 7′-dichlorodihydrofluorescine diacetate (DCFH-DA) for 1 h at 37 °C in the dark, to preload them with the DCHF-DA probe. They were then washed with PBS and resuspended in a 60Φ cell culture dish and exposed to F-mode, IZ + F mode or IZ + F + C mode treatment, with stirring for 120 min. After exposure, the cells were harvested and transferred to black cell culture fluorometric 96-well plates and their DCF fluorescence intensity was measured at excitation wavelength 480 nm, emission wavelength 530 nm and 530 nm cutoff. For DCF fluorescence images, bacterial suspensions were loaded on glass slides after incubation with DCFH-DA for 1 h, and exposed to F-mode, IZ + F mode and IZ + F + C mode, respectively, for 120mins. DCF fluorescence signals were detected with a confocal microscope (Olympus FV1200, Japan).

### Measurement of 8-Oxo-dG

To examine if the ions generated caused oxidative damage to bacteria, we analyzed oxidative damage to DNA. A sample (0.1 ml) of an approximately 10^8^ CFU/ml culture of *S. aureus* was filtered onto a 0.45 μm membrane filter, and exposed to the ionizer. After exposure, the bacteria were suspended in 5 ml of saline and sonicated briefly, and the suspension was centrifuged. DNA was extracted using a DNA extraction kit (iNtRON Biotechnology). The concentration of DNA was adjusted to 200 μg/ml by reading absorbance at 260 nm using a spectrophotometer (ND-1000, NanoDrop Technologies), and the formation of 8-oxodeoxyguanosine (8-Oxo-dG), a marker of oxidative DNA damage [12], was measured with an Oxiselect oxidative DNA damage ELISA kit (Cell Biolabs, Inc., San Diego, CA, USA).

### Assay of aconitase activity

We also analyzed aconitase activity, which is reported to be sensitive to oxidative damage. 0.1 ml of an approximately 10^8^ CFU/ml culture of *S. aureus* was filtered onto a 0.45 μm membrane filter, and exposed to the ionizer. After exposure, the bacteria were suspended in 5 ml of saline, resuspended by brief sonication and collected by centrifugation. They were then broken with a microbead beater, and enzyme activity was measured with an Aconitase Activity Colorimetric Assay Kit (Biovision) after adjusting concentrations of protein based upon absorbance at 562 nm (BCA Protein Assay Kit, PIERCE).

### Adenylate energy charge (AEC)

Cultures were serially diluted and deposited on 0.45 μm membrane filters by suction, then exposed to the various treatment modes. Preheated boiling buffer solution (50 mM Tricine, 10 mM MgSO_4_, and 2 mM EDTA at pH 7.8) was added to the samples and the mixtures were heated for 3 min. The boiled extracts were chilled on ice for a minimum of 10 min and kept at room temperature. To measure ATP, extracts were added to reaction buffer (75 mM Tricine, pH7.5; 5 mM MgCl_2_, and 0.0125 mM KCl). To measure ADP + ATP, extracts were added to the same reaction buffer with addition of 0.5 mM phosphoenolpyruvate (Sigma) and 0.4 μg/μl of pyruvate kinase (Sigma). AMP + ADP + ATP was measured by further addition of 0.5 μg/μl of adenylate (myo) kinase (Sigma) to the buffer. The ATP and ADP + ATP mixtures were incubated for 30 min at 30 °C, and the AMP + ADP + ATP mixtures were incubated for 90 min at 30 °C, and all were placed in a boiling water bath for 3 min to stop the reactions, chilled on ice and kept at room temperature. ATP was then determined with luciferin/luciferase using Luciferase Assay Reagent (Promega) and a SpectraMax L Luminometer (Molecular Devices). AEC was calculated as described by Atkinson [(ATP) + 0.5 (ADP)] / [(ATP) + (ADP) + (AMP)].

## Results

### Survival of bacteria after ionizer exposure

**Fig. 2:**
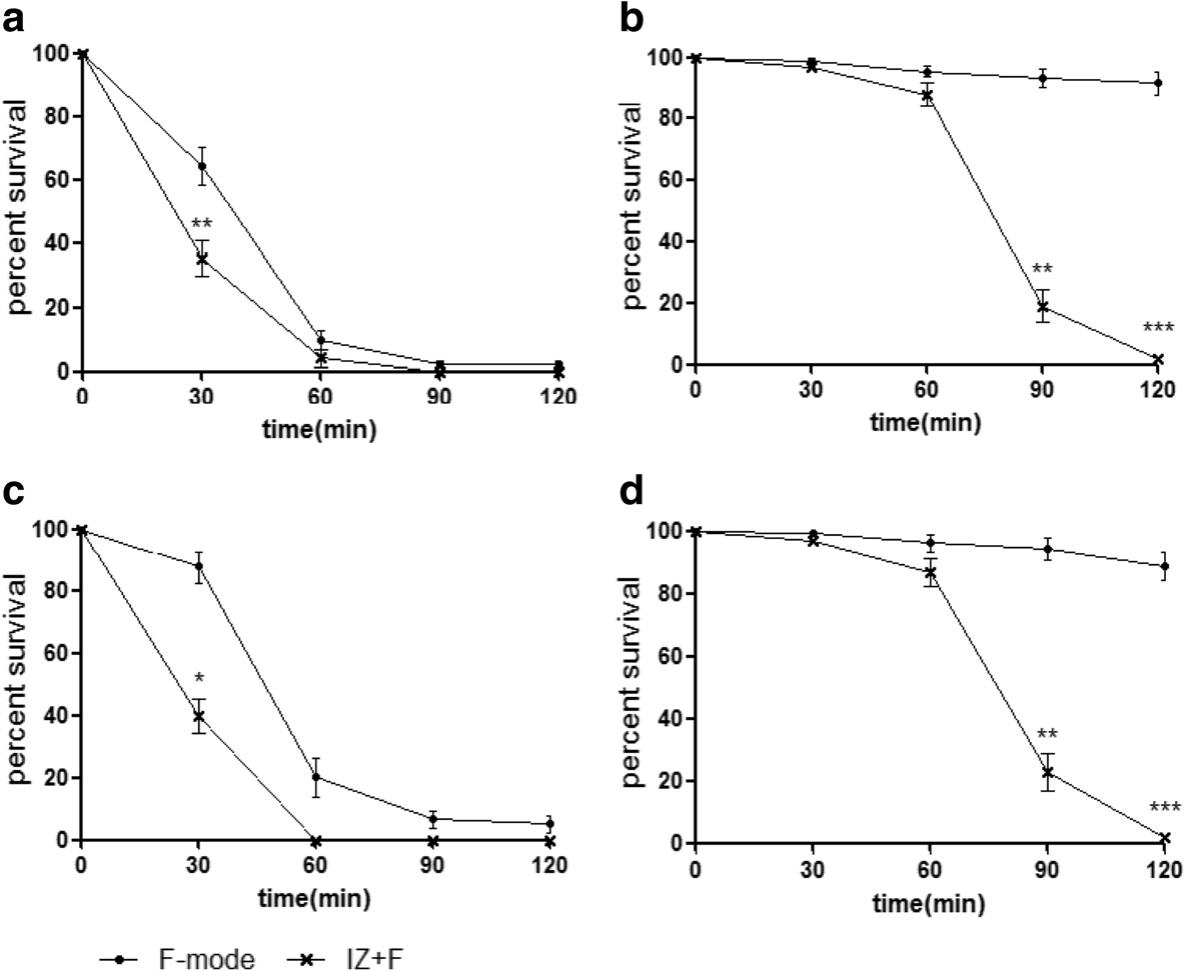
Survival after exposure to negative and positive ions. Each bacterial species was filtered and exposed to ion-with-fan (IZ + F) and fan only (F-mode) conditions. The percent survival shown are the means of 5 replicates in each experiment. **a** *E. coli*, **b** *E. faecalis*, **c** *B. subtilis*, **d** *S. aureus*

The negative and positive ion exposure experiments were performed in a sealed plastic chamber (46*46*46 cm, 100 L) at room temperature. An ionizer was installed on one of the walls of the chamber with a fan above to provide air. Bacteria on membrane filters (0.45 μm) placed in empty Petri-dishes without lids, were exposed to the ionizer as described above. The results of the ionizer exposure experiment are presented in Fig. 2, which shows survival verses exposure time for each bacterial species in F- versus IZ + F-mode. In the F-mode (fan only), there was considerable death of the *E. coli* and *B. subtilis* but not of *S. aureus* or *E. faecalis*. We assume that the *E. coli* and *B. subtilis* were sensitive to desiccating conditions. Exposure to the ionizer caused considerable death of *S. aureus* and *E. faecalis*. In this experiment we operated the fan to generate an air flow because in its absence survival of the bacteria was unaffected by the ionizer. These findings indicate that the negative and positive ions produced by the ionizer, or any ozone produced, have a bactericidal effect.

**Fig. 3:**
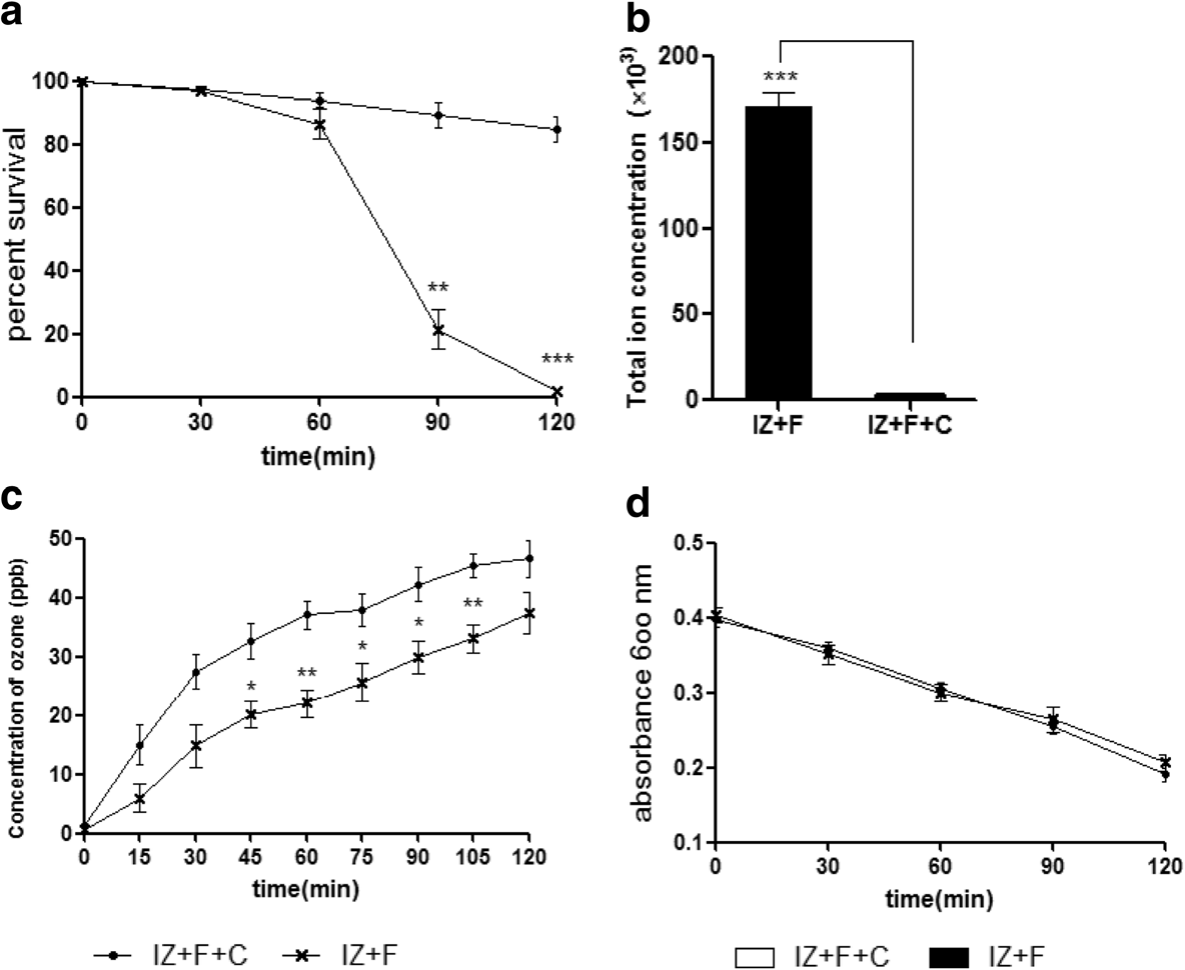
Effect of ion capture. **a** *S. aureus* was exposed to negative and positive ions with and without ion capture. The percent survival shown are the means of 5 replicates in each experiment. **b** The concentrations of ions measured with an ion meter during exposure to one ionizer with and without ion capture. **c** The concentrations of ozone measured with an ozone meter during exposure to one ionizer with and without ion capture. **d** The concentration of ozone was assessed from the discoloring of a solution of blue potassium indigotrisulfonate. Statistically significant differences are shown, ***p < 0.001

The ionizer generates ions by creating a corona discharge at the ends of the electrodes, and also generates ozone. The concentration of the ozone produced was monitored with an ozone meter (OZ 200 G), and was about 35 ppb after 2 h in the 100 L chamber (Fig. 3c). Although this was a relatively low concentration of ozone, it was important to assess its contribution to the bacterial killing. Therefore we removed negative and positive ions from the ionizer by covering it with a DC-operated voltage ion capture. Under these conditions the ion counter detected almost zero ions (Fig. 3b). We found that when the ionizer was covered with the ion capture, the concentration of ozone generated was also somewhat reduced. Therefore to generate a similar quantity of ozone in the presence and absence of the ion trap (IZ + F vs IZ + F + C mode), we raised the input power of the ionizer to 14 V in the IZ + F + C mode. The concentration of ozone was monitored by ozone meter or the indigo method to confirm that the concentration of ozone generated in the IZ + F + C mode were not lower than that of IZ + F mode (Fig. 3c, and d). As shown in Fig. 3a, ion capture dramatically reduced the bactericidal effect of the ionizer, indicating that the ozone generated had little or no bactericidal effect under these conditions, and that the ions produced were responsible for almost all the bacterial killing.

### ROS

Since we had shown above that the ionizer that we used generated only small amounts of ozone making an insignificant contribution to the bactericidal effect, we attempted to determine the basis of the bactericidal effect of the ions. In the following experiments, we used *S. aureus* only, as it was relatively stable under our control conditions (F- mode).

**Fig. 4:**
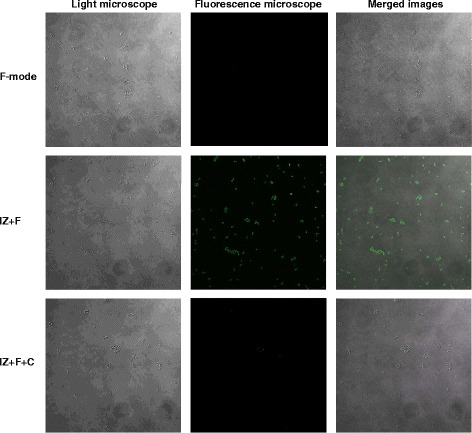
Generation of ROS examined with a fluorescence microscope. *S. aureus* suspensions were spread on glass slides after incubation with DCFH-DA for 1 h, and exposed to the F-mode, IZ + F-mode and IZ + F + C-mode for 120mins; they were then examined with a fluorescence microscope (1,000X, confocal microscope, Olympus FV1200)

**Fig. 5:**
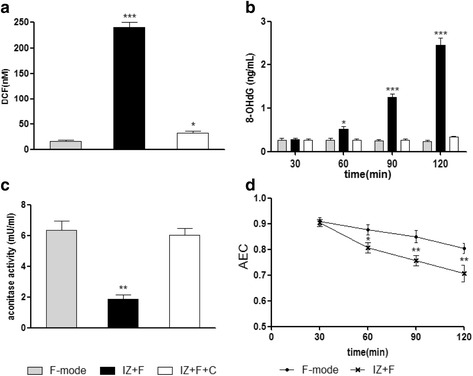
Generation of ROS by the ionizer and their effects on *S. aureus*. **a** *S. aureus* suspensions were incubated with DCFH-DA for 1 h, washed with PBS, and exposed to the three modes as above and DCF fluorescence intensity was measured. **b** Formation of 8-hydroxydeoxyguanosine (8-OHdG) in *S. aureus* after exposure to the three modes. **c** The effect of ion exposure on aconitase activity. Bacteria were exposed to the three modes for 120 min. **d** Resulting levels of adenylate energy charge [(ATP) + 0.5(ADP)] / [(ATP) + (ADP) + (AMP)]. Each experiment was repeated at least 5 times. Statistically significant differences are shown. *p < 0.05, **p < 0.01, ***p < 0.001

It has been reported that the life times of negative ions (O2^−^, N2^−^) and positive ions (O2^+^, N2^+^) are as short as microseconds, and that they can be extended to 1–2 min in the form of clusters [13–15]. We thought that if the ionizer’s ions could persist for 1–2 min in this way, they might behave as ROS. Free radicals and other reactive species are produced by living organisms and can damage biomolecules [10, 11, 16]. Oxidative stress induced by exposure to negative and positive ions could therefore result in the production of intracellular reactive oxygen species. To examine this possibility DCFH-DA-loaded bacteria were exposed to negative and positive ions (IZ + F mode). A substantial increase of intracellular ROS was detected with a fluorescence microscope (Fig. 4), and fluorometer (Fig. 5a). These findings show that exposure of bacteria to negative and positive ions leads to oxidative stress as shown by the increased intracellular ROS. Less than 10 % of these ROS were detected in the ion-capture mode.

### Oxidative DNA damage

Since ROS were detected in *S. aureus* exposed to the ionizer, we examined the well-known targets of ROS. When an *S. aureus* suspension was exposed to ions generated by the ionizer, the level of 8-oxo-dG, an oxidized derivative of deoxyguanosine, increased dramatically with time (Fig. 5b). By contrast, air flow by fan (F-mode) or the ion-capture mode (IZ + F + C mode) did not elevate 8-oxo-dG.

### Aconitase

Enzymes containing catalytic [4Fe-4S] clusters, such as aconitase, are thought to be important targets of ROS [17]. We prepared cell-free extracts of *S. aureus* exposed to the ionizer, and found that the activity of aconitase decreased by about two-thirds after ion exposure, as expected if ROS were being produced (Fig. 5c).

### Energy charge values

Energy charge is considered a metabolic control parameter. Viability is maintained at values of adenylate energy charge between 0.8 and 0.5, and cells die at values below 0.5. [18–20] We measured the effect of the ionizer on adenylate energy charge. In fan only mode, the adenylate energy charge was about 0.85 (Fig. 5d). In IZ + F mode the energy charge fell to about 0.7.

## Discussion

There has been much interest in the use of air ionizers to control infections in the air or on surfaces, and there have been a number of studies of their killing effects on bacteria and fungi [1–5]. However, it has not been clear to what extent the effects of ionizers are due to the ions or to the ozone produced [8].

In our experiments we used a newly developed ionizer that produces relatively small amounts of ozone (Fig. 3). The plasmaster 2.5G that we used generated about 35 ppb of ozone over 2 h in 100 L; however, in previous reports using ionizers ozone concentrations were reported to be from 100 ppb (8) to 2.3 ppm (3). The concentrations of ozone and ions are affected by the volume of the chamber and the closeness of the generator and detector. In the conditions we used, the results showed that very little of the bactericidal effect of this ionizer was due to ozone because ion capture virtually abolished it. We were therefore able to analyze how ions, specifically, exert their effects on bacteria.

In previous studies most cell killing was examined on sessile cells on agar plates. With the ionizer used in this study, however, it was not easy to detect any effects in a 100 L chamber over two hours of exposure. When we used the plasmaster 2,5G on sessile cells, although *Bacillus subtilis* survival was reduced to less than 10 % after 2 h, the other 3 strains tested remained unaffected over 2 h (Additional file 1: Figure S1). We were able to detect some killing of the other bacteria in a 10 L chamber equipped with 4 ionizers and operated for one hour, but we did not use these conditions because we were unable to eliminate the effect of ozone. Instead, we filtered the bacteria onto membrane filters (0.45 μm, containing 200 μl of a 1:100 dilution of LB), and exposed them in a 100 L chamber equipped with an ionizer, and with a fan to provide air flow (1.5 m/s). Under these conditions we found that the bacteria tested were killed, as seen in Fig. 2 (and we also tested for several yeasts including*, Candida albicans, Candida vartiovaarai,* and *Cryotococcus flavus* to get the results of 20–30 % of the cells survived after 2 h exposure under these experimental conditions; Additional file 2: Figure S2), The tested bacteria were not killed when the fan was turned off, but as we used the fan alone (no ionizer) mode to compare, it was clear that the ions had specific effects on the bacteria.

Previous studies have demonstrated that negative and positive ions have a bactericidal effect. There are several suggested mechanisms for the action of ionizers: electrical phenomena (ions, electric charge, ozone production, [3]) and electrostatic repulsion [7]. Our evidence that negative and positive ions generate oxidative stress points to an oxidizing effect. In previous studies, the physical and biological mechanisms underlying the effects of ions remained unclear. To clarify these mechanisms we examined the production of ROS by the ionizer, as well as the effect of the ROS on the bacteria. As shown in Fig. 5, the ionizer did indeed generate ROS, and the ROS caused oxidative damage to the bacterial DNA, as shown by a marked increase in the level of 8-oxo-dG, as well as a pronounced reduction in the activity of aconitase, which is known to be sensitive to ROS [21]. The ROS generated by the ionizer also reduced the adenylate energy charge of the cells, although the AEC was not reduced to the level that leads on its own to cell death. Fumarase activity was not affected by the ionizer (data not shown). These findings indicate that negative and positive ions induce oxidative stress, which causes cell death by oxidative damage to cells.

## Conclusions

The newly developed ionizer, which generates a relatively low concentration of ozone, showed a bactericidal effect. The generated negative and positive ions induced oxidative stress on the bacteria during exposure. Our results indicate that negative and positive ions induce oxidative stress, which causes cell death by oxidative damage to cells including damage to DNA. Our findings suggest a potential mechanism of the ions on bactericidal effect.

## Abbreviations

8-OHdG, 8-hydroxydeoxyguanosine; AEC, adenylate energy charge; DCFH-DA, dichlorodihydrofluorescine diacetate; F-mode, fan only mode; IZ + F + C mode, ionizer, fan and ion capture mode; IZ + F-mode, ionizer and fan mode; ROS, reactive oxygen species

## Additional files

Additional file 1: Figure S1. Survival of sessile cells on agar plates after exposure to negative and positive ions. Each bacterial species was spread on agar plates (NA medium) and exposed to ions with fan (IZ + F) and fan only (F-mode) conditions. The percent survival shown are the means of 5 replicates in each case. (A) *E. coli*, (B) *E. faecalis*, (C) *B. subtilis*, (D) *S. aureus*. (PDF 34 kb) Additional file 2: Figure S2. Survival of yeasts after exposure to negative and positive ions. Each yeast species was exposed on membrane filters to ions-with-fan (IZ + F) and fan only (F-mode) conditions. The percent survival shown are the means of 3 replicates in each experiment. (A) *Candida albicans,* (B) *Candida vartiovaarai,* (C) *Cryotococcus flavus.* (PDF 32 kb)
