# Supplementary figures and images for: The bactericidal effect of an ionizer under low concentration of ozone
Source: BMC Microbiol. 2016 Jul 30;16:173. doi: 10.1186/s12866-016-0785-5 (PMC4967512; doi:10.1186/s12866-016-0785-5)

(A)

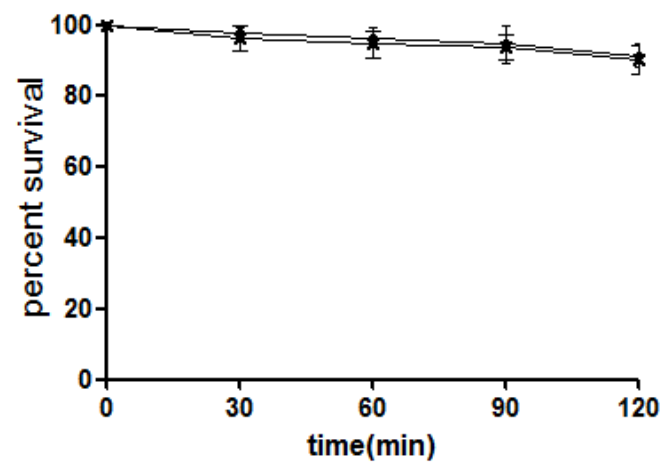

(B)

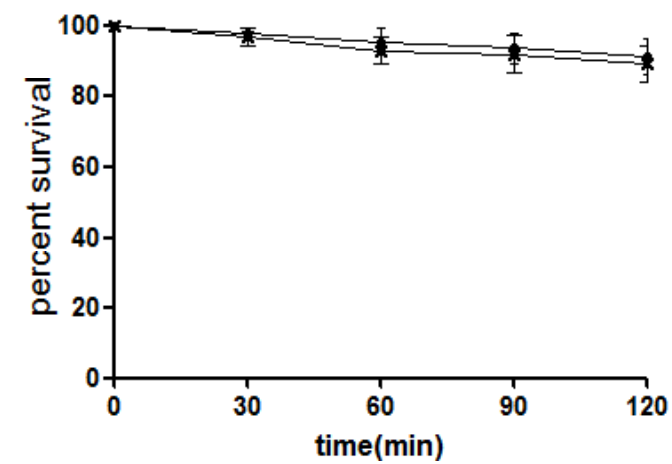

(C)

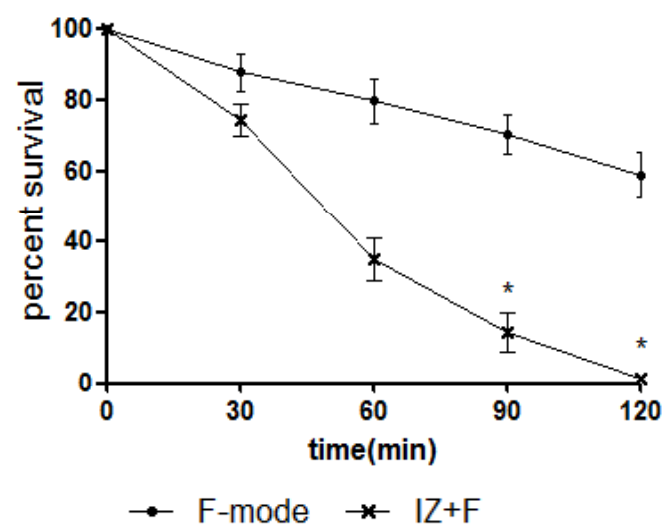

(D)

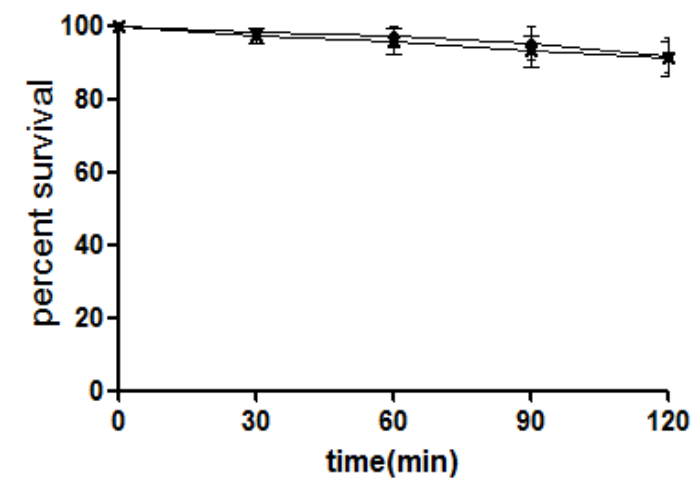

Supplement: Additional file 1: Figure S1. — Survival of sessile cells on agar plates after exposure to negative and positive ions. Each bacterial species was spread on agar plates (NA medium) and exposed to ions with fan (IZ + F) and fan only (F-mode) conditions. The percent survival shown are the means of 5 replicates in each case. (A) E. coli, (B) E. faecalis, (C) B. subtilis, (D) S. aureus. (PDF 34 kb) [file 12866_2016_785_MOESM1_ESM.pdf]

(A)

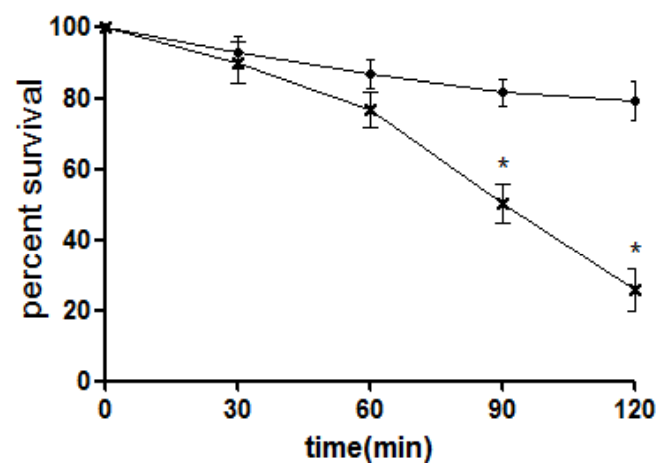

(B)

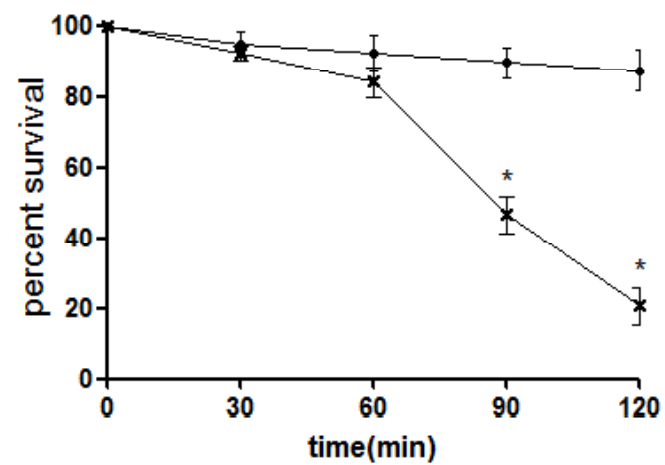

(C)

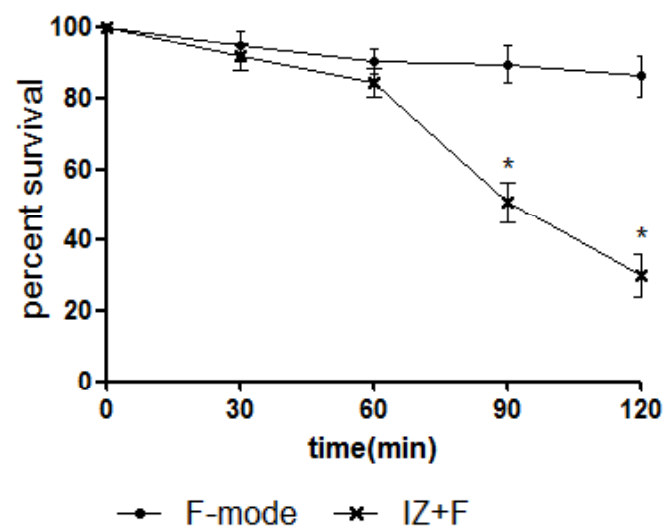

Supplement: Additional file 2: Figure S2. — Survival of yeasts after exposure to negative and positive ions. Each yeast species was exposed on membrane filters to ions-with-fan (IZ + F) and fan only (F-mode) conditions. The percent survival shown are the means of 3 replicates in each experiment. (A) Candida albicans, (B) Candida vartiovaarai, (C) Cryotococcus flavus. (PDF 32 kb) [file 12866_2016_785_MOESM2_ESM.pdf]
